# Concomitant intramyocardial and hepatic hydatid cysts diagnosed by multi-modality imaging: A rare case report

**DOI:** 10.3389/fcvm.2022.1055000

**Published:** 2022-12-14

**Authors:** Hoai Thi Thu Nguyen, Viet Tuan Pham, Hung Duc Duong, James N. Kirkpatrick, Walter Robert Taylor, Hung Manh Pham

**Affiliations:** ^1^Vietnam National Heart Institute, Bach Mai Hospital, Hanoi, Vietnam; ^2^Department of Internal Medicine, VNU-University of Medicine and Pharmacy, Hanoi, Vietnam; ^3^Cardiovascular Division, Department of Medicine, University of Washington Medical Center, Seattle, WA, United States; ^4^Department of Bioethics and Humanities, University of Washington Medical Center, Seattle, WA, United States; ^5^Mahidol Oxford Tropical Medicine Research Unit, Bangkok, Thailand; ^6^Centre for Tropical Medicine and Global Health, University of Oxford, Oxford, United Kingdom; ^7^Department of Cardiology, Hanoi Medical University, Hanoi, Vietnam

**Keywords:** hydatid cyst, cardiac echinococcosis, intramyocardial hydatid cyst, three-dimensional echocardiography, multi-modality imaging

## Abstract

Cardiac echinococcosis is a potentially fatal form of hydatid disease; yet, its diagnosis and treatment are challenging due to the variability in its clinical manifestations and due to its various unpredictable preoperative complications. Multi-modality imaging is shown to provide important guidance for the treatment and decision-making. We report a rare case of a 50-year-old woman who had concomitant cardiac and hepatic hydatid cysts. She presented with abdominal pain and elevated eosinophilic white blood cells. The initial abdominal ultrasound and computerized tomography revealed a large cyst in the liver. An intramyocardial cyst was detected by two-dimensional echocardiography. Three-dimensional echocardiography increased the confidence level of two-dimensional echocardiography by displaying the three-dimensional volume of the cyst and allowing visualization of its spatial characteristics and the relationships with adjacent cardiac structures, which was subsequently confirmed at surgery. Multi-detector computed tomography and magnetic resonance imaging helped localize and define the typical morphological features of the cyst. Serology and antigen detection were used for diagnosis. This rare case underlines the integration of clinical, multi-modality imaging, and pathological data in the diagnosis of concomitant intramyocardial and hepatic hydatid cysts. Surgical resection of cysts and anthelmintic medication were successful in the management of this patient.

## Introduction

Echinococcosis in humans occurs as a result of infection by the larval stages of the taeniid cestodes of the genus *Echinococcus*. When present, hydatid involvement often manifests as cystic lesions in many organs (1), which can be symptomatic due to expansion and mass effect or due to rupture that occasionally leads to anaphylaxis and death. Cardiac hydatid cysts are found in fewer than 2% of cases of hydatidosis (2). The diagnosis is challenging because of the long latency between the infection and the manifestation of the disease (3). Decision-making regarding therapy for cardiac hydatid cysts depends on their locations, size, hemodynamic influences, and the risk of rupture. Two-dimensional (2D) and three-dimensional (3D) echocardiography, multi-detector computed tomography (MDCT), and magnetic resonance imaging (MRI) can show the cystic nature of the mass and its relation to the cardiac chambers.

In this article, we report a rare case of a female patient who presented with abdominal pain and elevated eosinophilic white blood cells. Multi-modality imaging showed an unexpected intramyocardial hydatid cyst and a large hepatic cyst with a definitive diagnosis confirmed by pathology of samples that were acquired during surgery.

## Case presentation

A 50-year-old female farmer from a Northern Midland province of Vietnam complained of right upper quadrant and epigastric pain, which was described as a dull ache that had been present for 2 months. In addition, she experienced an intermittent “stinging” sensation in the chest. She was otherwise well and denied weight loss, malaise, fever, rash, and gastrointestinal or respiratory symptoms. Her medical history was normal. She had many dogs as pets in her house. Findings on physical examination were unremarkable. The 12-lead electrocardiogram (ECG) with sinus rhythm at 76 beats per minute and 24-h ambulatory ECG were normal. A chest X-ray examination showed a normal cardiothoracic index and clear lung fields. The patient had eosinophilia (eosinophilic white blood cell 22.1%) in association with a mild increase in plasma liver enzymes (AST:39U/L, ALT: 56U/L) and the erythrocyte sedimentation rate (1h/2h:30/36). Other blood and urine test results were normal.

Abdominal ultrasound demonstrated an echo-lucent lesion measuring 150 × 80 mm in the right lobe of the liver. An abdominal computed tomography scan with contrast was performed to assess the tissue characteristics, size, and evidence of local complications and detect other visceral cysts. The cyst was described as an encapsulated structure measuring 115 × 86 mm, with a thin capsule containing homogeneous liquid. The biliary tract was normal on both abdominal ultrasound and CT scan. Cerebral and pulmonary cysts were ruled out by head and chest CT scans, respectively.

**Figure 1:**
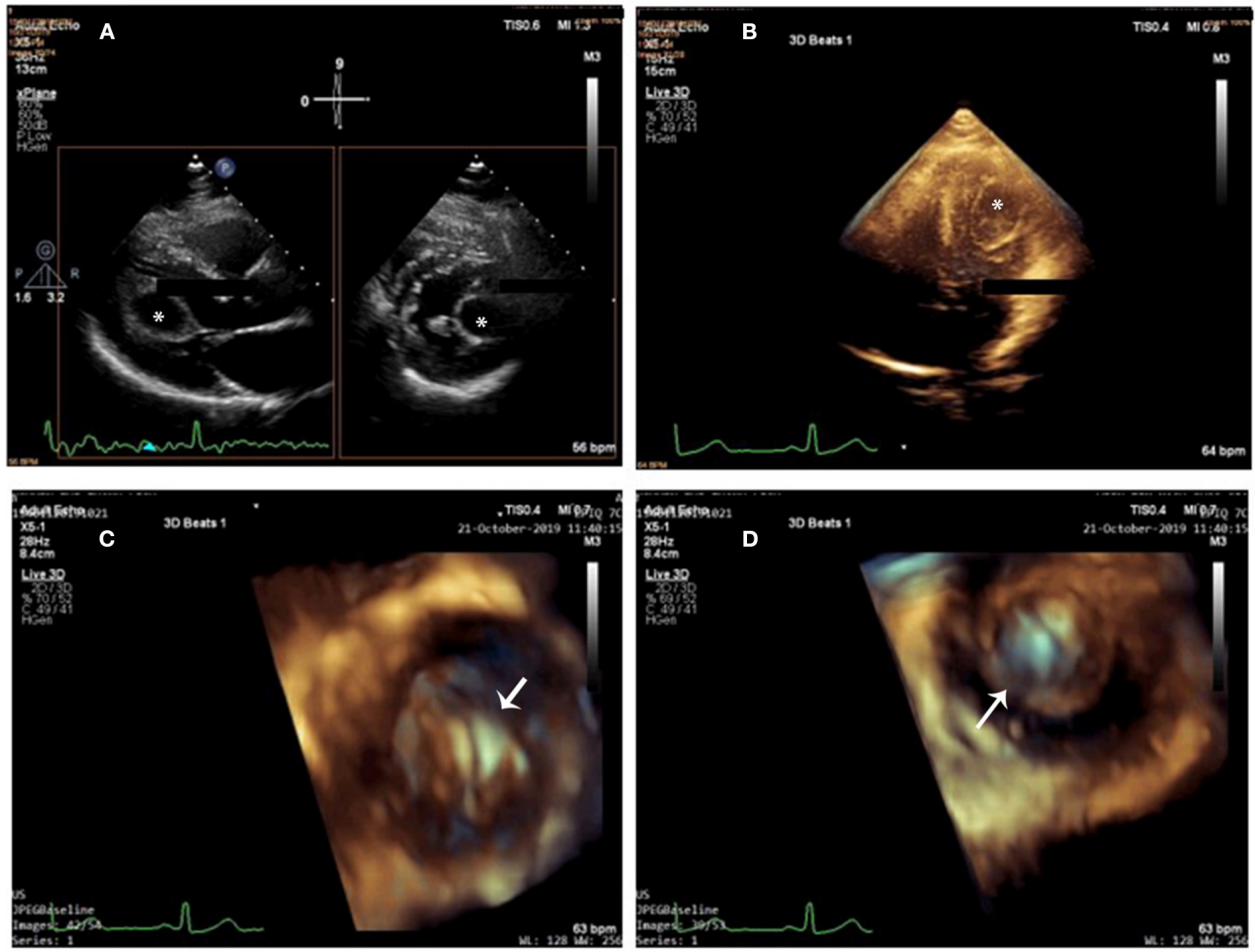
Cardiac cyst on 2D-TTE and 3D-TTE. **(A)** 2D X-plane imaging showed a drop-like and echo-lucent intramural structure (star) bulging into the left ventricle from the myocardium of the left ventricular lateral wall. **(B)** 3D full-volume imaging: The cyst was round and unilocular, with liquid content and well-defined edges (star). **(C,D)** 3D zoom imaging (en-face view) from outside **(C)** and from inside **(D)** perspectives (arrow), the cyst wall looked homogeneously smooth and hyper-echogenic. Cystic sludge was hypo-echogenic.

Transthoracic echocardiography (TTE) revealed a drop-like and echo-lucent intramural structure measuring 29 × 21 mm, bulging into the left ventricle from the myocardium of the left ventricular lateral wall and moving synchronously with the cardiac cycle (Figure 1A). On Doppler echocardiography, no color flow was observed within the cystic cavity. Neither the aortic valve nor the mitral valve was influenced by the cyst. The cardiac cyst did not cause intracardiac obstruction. Left ventricular (LV) and right ventricular (RV) dimensions and function and systolic pulmonary artery pressure were normal. These findings were compatible with the patient's clinical functional class (New York Heart Association Classification I).

Three-dimensional transthoracic echocardiography (3D-TTE) was performed to image the cyst in multiple planes. In total three different modalities of 3D echocardiography, including live 3D (narrow-angle), 3D zoom, and full volume (wide angle), were acquired and then cropped to visualize the cyst using “en-face” views. The cyst was round and unilocular, with liquid content and well-defined edges; its wall looked homogeneously smooth and hyper-echogenic when seen from outside and inside (Figures 1B–D and Supplementary Videos 1–3). Cystic sludge was hypo-echogenic. The 3D dataset was used to measure cyst size (28 × 21 × 22 mm), and color Doppler confirmed that it was not vascularized. Enhanced description of the cyst with 3D-TTE helped in the differential diagnosis and aided in the understanding of the surrounding structures. There were no mitral prolapse, no mitral annular dilation, and no mitral regurgitation seen on 3D-TTE.

**Figure 2:**
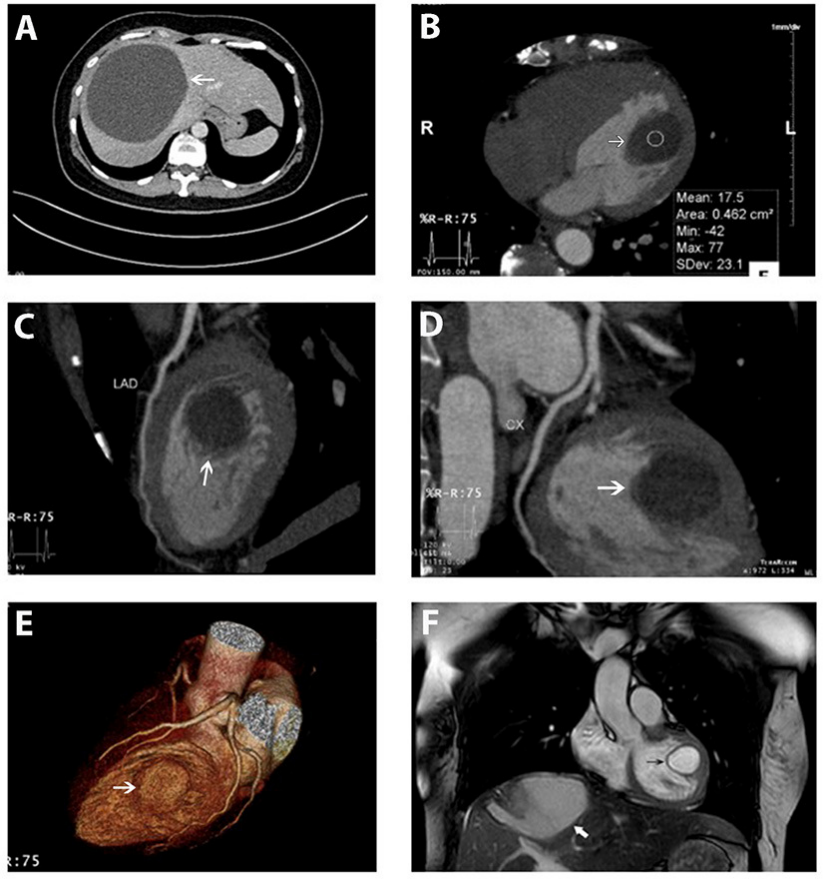
Hepatic cyst and the cardiac cyst on CT and on MRI. **(A)** A giant hepatic cyst was seen on the abdominal CT scanner (white arrow). **(B–E)** An encapsulated, rounded, and fluid-attenuated non-calcified structure along the LV lateral wall (white arrow) and its spatial relationship with coronary arteries (left anterior descending, circumflex) on MDCT. **(F)** Cardiac cyst (black arrow) and the hepatic cyst (white arrow) were simultaneously depicted on MRI. The cardiac cyst was a single intramuscular cyst located at the anterolateral papillary muscle attached to the LV lateral free wall, protruding into the LV cavity, not enhanced by the gadolinium. Myocardial perfusion and enhancement were normal.

On cardiac CT images, the lesion looked primarily intracavitary. Contrast-enhanced MDCT of the heart showed an encapsulated, rounded and fluid-attenuated, non-calcified structure along the LV lateral wall measuring 32 mm × 24 mm, its morphologic features looked similar to that of the hepatic cyst which was shown in the Figure 2A. There was no enhancement seen following IV contrast. Coronary MDCT excluded coronary stenosis; coronary aneurysm, which may have mimicked some of the findings on echo; and coronary compression caused by the cyst. In computed tomographic 3D volume-rendered images, the cyst was also clearly seen not in contact with coronary arteries (Figures 2B–E).

Cardiac MRI also demonstrated a single intramuscular cystic mass located at the anterolateral papillary muscle attaching to LV lateral free wall and protruding into the ventricular cavity (Figure 2F and Supplementary Video 4). The cyst was not enhanced by gadolinium and was measured at 24 × 23 × 30 mm. Myocardial perfusion and enhancement were normal. LVEF and cardiac indexes were 77% and 2.8 l/min/m^2^, respectively. The flow void on CMR confirmed the normal activity of the cardiac valves without intracardiac obstruction.

**Figure 3:**
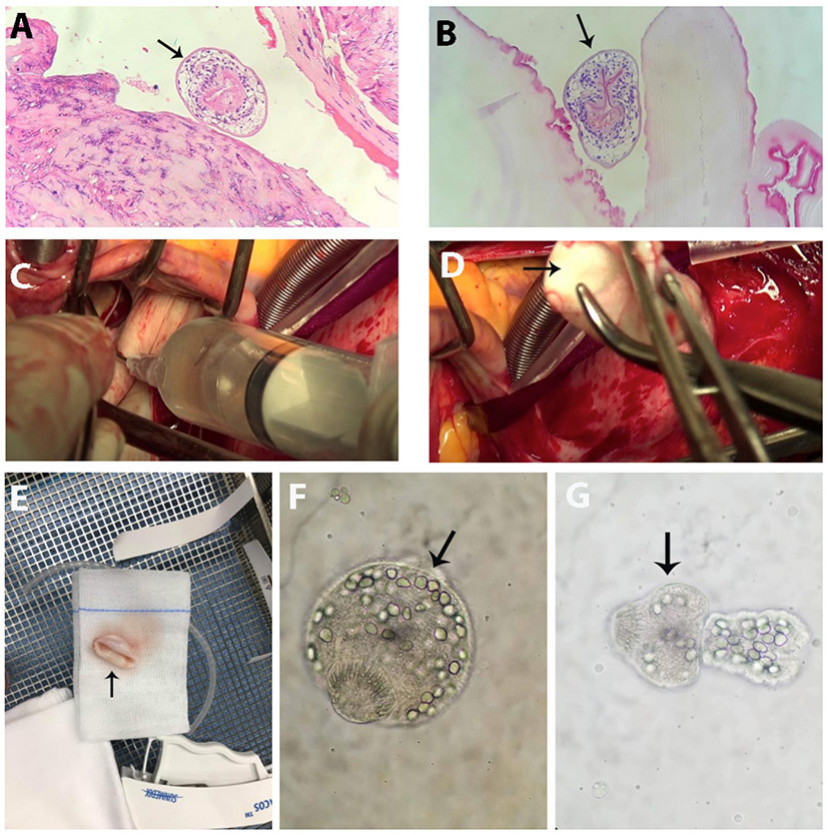
Surgical findings and Echinococcus larvae. **(A,B)** Echinococcus larvae (arrow) in the hepatic cyst specimen. **(C)** Cardiac cyst fluid that looked clear yellow was aspirated. **(D)** Opalescent white and smooth sphere cyst (arrow) was totally removed from the heart. **(E)** Cyst specimen (arrow). **(F,G)** Echinococcus larvae at different stages (arrow) in the cardiac cyst fluid.

We performed serological enzyme-linked immunosorbent assay (ELISA) tests for *Entamoeba histolytica, Strongyloides, Toxocara*, and *Echinococcus* and examined stool for the eggs of *Fasciola hepatica*. All results were negative. Because the hepatic cyst was very large and appeared to be at risk for rupture, surgical removal was attempted. However, only a partial cystectomy was possible because it was located too deeply in the liver tissue. The experienced surgeons resected the tip of the cyst and drained all the fluid inside. *Echinococcus* larvae were found in the histological specimen of the hepatic cyst wall (Figures 3A,B). She was treated with albendazole 400 mg b.i.d for 8 weeks before the cardiac surgery.

Since there were concerns for cyst rupture because of the mechanical contractility of the left ventricle, cardiac surgery was undertaken by the experienced cardiothoracic surgeons in the cardiovascular operating room to remove the cyst under cardiopulmonary bypass to minimize the risk of cyst leakage. The anterior papillary muscle containing the cyst was pale and swollen, and on exposure, the cyst was an opalescent white and smooth sphere containing clear yellow liquid (Figures 3C–E). The fluid was aspirated completely. Intravenous solumedrol was applied to the cyst to prevent an allergic reaction. The cyst cavity was excised intact, and the affected papillary muscle was reattached. The cystic fluid contained larvae at different stages that had specific morphological characteristics of *Echinococcus granulosus*, according to the designation of the World Health Organization and the World Organization for Animal Health (Figures 3F,G) (4).

**Figure 4:**
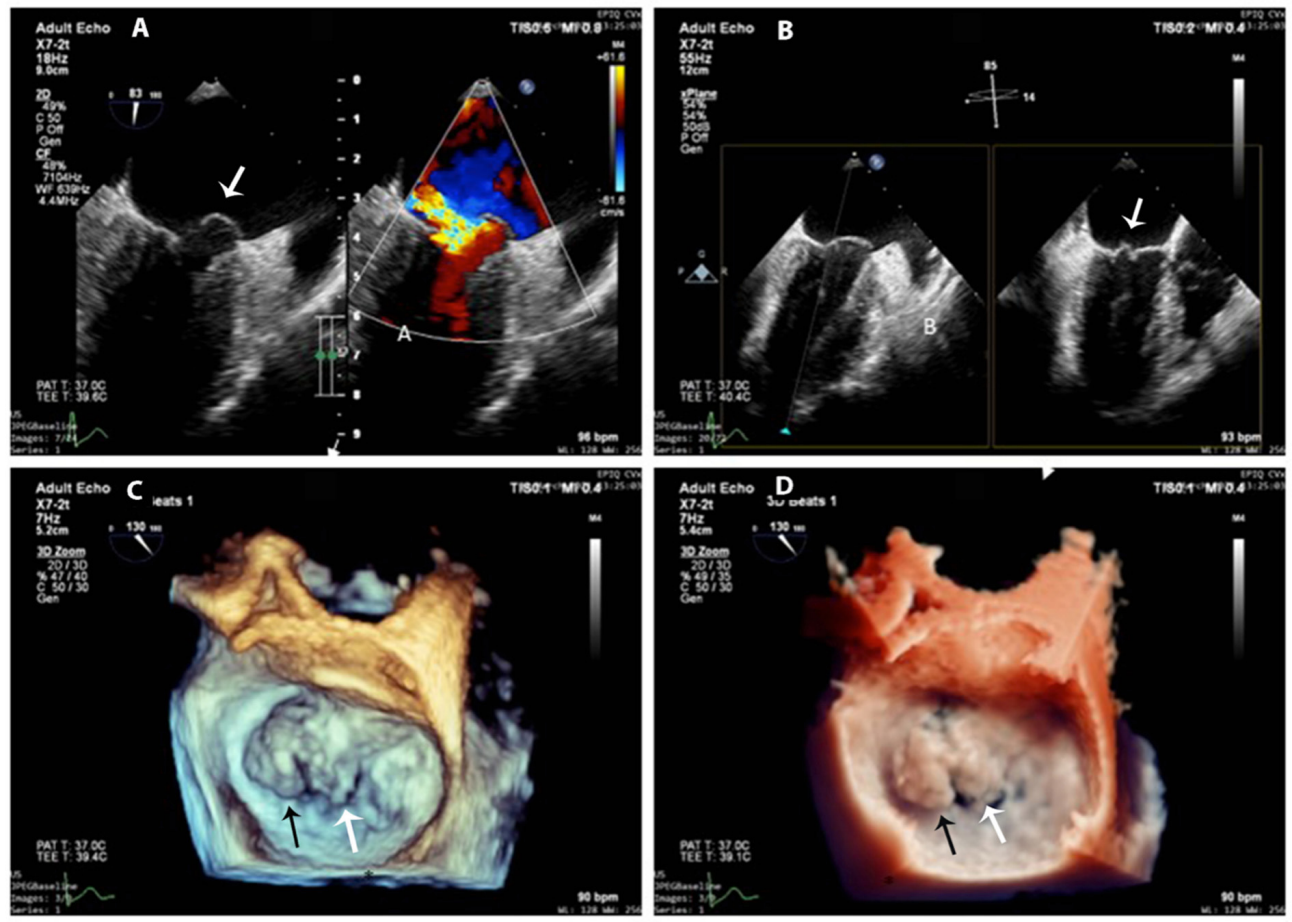
3D-TEE at 1-year follow-up. **(A)** Mitral anterior leaflet prolapse (white arrow) on a mid-esophageal two-chamber view (left panel) and the origin of the regurgitation jet with color Doppler (right panel). **(B)** X-plane imaging with two orthogonal views shows exactly the prolapsed scallops (white arrow). **(C,D)** 3D-zoom images of the mitral valve from the left atrial perspective (surgeon's en-face view) show exactly A1 (black arrow) and A2 (white arrow) prolapse **(C)** normal 3D gain vs. **(D)** true view mode.

After the cardiac cystectomy, the eosinophilic count decreased significantly (13.1% at 4 days post-operation). Post-operative TTE showed an LV cavity devoid of masses, normal LV systolic function, and trivial mitral regurgitation. The patient continued receiving albendazole 200 mg b.i.d for 2 more weeks. Post-operative 12-lead and 24-h ambulatory ECGs were normal. At 1, 6, and 12 months after cardiac surgery, the patient felt well-without symptoms, and her blood tests, abdominal ultrasound, and echocardiographic LV and RV systolic functions were normal. However, the 12-month 2D-TTE revealed mild to moderate mitral regurgitation due to prolapsed A1 and A2 segments that were confirmed by 3D transesophageal echocardiography (3D-TEE) (Figures 4A–D); the prolapse was probably due to resection of the anterior papillary muscle at the time of cyst removal. The 3D-TTE and 3D-TEE showed no abnormal structures in the LV cavity, and the LV dimensions and ejection fraction were within normal limits and without any significant changes from the previous echo studies.

## Discussion

Cystic echinococcosis, caused by *Echinococcus granulosus*, is common in pastoral areas. In Southeast Asian countries such as Thailand, Vietnam, and Indonesia, *Echinococcus* is very rare (5). In Vietnam, only two cases of cystic lung echinococcosis have been previously reported (6). The liver is the most common site, ~60%, followed by the lungs, ~20%. Hydatid cysts can be found in the pancreas, spleen, pelvis, rectum, kidneys, urinary tract, central nervous system, musculoskeletal system, bone, and skin (7–10). Cardiac hydatid cysts are rare, accounting for fewer than 2% of reported cases; the prevalence of asymptomatic cardiac hydatid cysts is even rarer (2). In Vietnam, our patient was the first cardiac echinococcosis case to be reported.

*Echinococcus granulosus* is a cyclophyllid cestode and is also known as the hydatid worm or dog small tapeworm. The adult worms reside and reproduce in the small intestines of dogs and wild carnivores, defining them as the definitive hosts. Humans and other animals are intermediate hosts (i.e., they harbor the larval stage or asexual forms). They become infected by ingestion of eggs passed in dog feces, and this leads to the development of hydatid cysts. During primary infection, eggs may reach the heart through the lymphatic system or *via* the coronary circulation. Alternatively, cysts in the liver and lungs may rupture and release daughter cysts, larvae, and fragments of the germinal layer, which then settle in the myocardium to produce new cysts. The coronary artery anatomy explains the cardiac distribution of cysts; the most common cardiac location is the left ventricular wall (60%) because the left ventricle receives the largest amount of the coronary blood supply, followed by the right ventricle (10%), interventricular septum (4%), left atrium and left atrial appendage (6%), pulmonary artery (6%), pericardium (7%), and right atrium (3%) (11). Cysts may localize in the ventricular wall, outflow tract, or apex or may spread from the left ventricular wall to the ventricular septum. The pericardium may also be affected, causing acute pericarditis that may lead to constrictive pericarditis over time. By contrast, the endocardium does not react to the presence of hydatid cysts and, therefore, may be more easily invaded by the cyst. Cardiac hydatid cysts are often part of multiple organ involvement (1, 5). Patients may be asymptomatic for many years, while in other patients, symptoms may develop when there is a mechanical obstruction, for example, coronary artery compression, or if the conduction system is compromised by the expanding cyst. Serious complications include cyst rupture leading to acute anaphylactic shock and death, cardiac tamponade also due to a rupture or an expanding cyst, cerebral or peripheral arterial embolism, acute coronary syndrome, arrhythmias, ventricular dysfunction, valvular regurgitation, and sudden unexplained death (12–17).

Electrocardiography may reveal Q-wave or inverted T-wave in leads overlying the affected myocardium or may disclose evidence of conduction abnormalities (12, 13).

2D-TTE is the first-line diagnostic method of choice for cardiac hydatid cysts. It is rapid and portable and can indicate whether the cyst is enclosed or ruptured, whether it communicates with the blood supply within the heart, and whether it is vascularized. Echocardiography can evaluate the cyst mobility, attachment, and hemodynamic consequences and follow the results of treatment. Patients with exposure to or with the presence of echinococcosis in any part of the body should undergo an assessment by echocardiography as part of “staging” this multisystem disease. Our patient had concomitant cardiac and hepatic hydatid cysts but had only abdominal symptoms. She experienced no dyspnea, palpitations, or cough. A lack of cardiovascular signs and symptoms was likely related to the size and the location of the cardiac cyst, making the diagnosis of cardiac hydatid disease difficult. Thus, routine TTE plays an important role in screening for this potentially fatal condition. Mass population screening of cystic *Echinococcus* in endemic areas using portable ultrasound is considered the best method for early diagnosis, especially in asymptomatic cases (9, 10).

The use of live/real-time 3D echocardiography is the newest approach in the assessment of intracardiac cysts, which helps image the cysts in multiple planes from outside and from inside and allows for a volumetric assessment of a cyst over a linear measurement as is obtained with 2D imaging. 3D echocardiography showed the texture of the cyst and helped confirm the continuity of the cyst to the left ventricular wall by visualization in multiple planes. Full-volume images can be obtained by 3D echocardiography and sectioned in multiple planes to examine and assess intracardiac cysts in terms of their homogeneous or heterogeneous nature, point of attachment, vascularity, and calcifications. The tissue characteristics of the cysts are peculiar to 3D echocardiography, which helps in the determination of their etiology and guides surgical planning. This information is vital, particularly since surgical cyst excision is the treatment of choice for this rare, but life-threatening, disease.

MDCT and MRI provide important details regarding the size, composition, presence of daughter cysts, anatomical location, and extracardiac involvement (9). Contrast-enhanced CT and MRI are helpful for localizing and defining the morphological features of hydatid cysts. MDCT can evaluate the coronary artery system and differentiate cysts from vascular structures. CT is also the best method for detecting cyst wall calcification. MRI is used for detecting tiny cysts, which might be missed when using ultrasound. MRI of a hydatid cyst demonstrates a hypointense lesion on T1-weighted images and a hyperintense lesion on T2-weighted images. The cyst has a defined peripheral wall because of the fibrous content of the pericyst and, therefore, may exhibit mild enhancement on delayed contrast-enhanced CT and MR images, displayed as ring enhancement. Our patient's cyst had no internal enhancement because its contents were avascular. To investigate LV or RV outflow tract obstruction and the status of coronary arteries, cardiac catheterization and angiography may be useful but can cause the rupture of the cyst. Angiography and scintigraphy may be helpful in diagnosing pulmonary hydatid embolism.

For cardiac differential diagnosis, we had to consider myxoma, fibroma, angiosarcoma, lipoma, and teratoma. Myxomas are typically found within the atria, are often pedunculated and attached to the atrial septum, are only rarely found in the left ventricular cavity, and have discrete echo-lucencies representing necrosis/hemorrhage. Fibromas are principally embedded in the myocardium and often manifest in children. Angiosarcomas are highly vascularized and tend to be located in the right atrium. Lipomas can be detected at any age and are seen in the left ventricle, atrial septum, and right atrium; they may also be resident in the subendocardium.

Eosinophilia is presented in <25% of patients with echinococcal cysts due to spontaneous cyst leakage or occult intrabiliary rupture (7). ELISA is the most serological method most commonly used. Sensitivity is high (80–94%) for hepatic hydatid and low for lung hydatid (65%) cysts. Sensitivity is reduced with cysts in certain sites (e.g., brain and eye) and in early and late inactive cysts. Therefore, negative serology does not exclude the diagnosis, as in our patient. Test specificity may be low because of cross-reaction with other parasitic infectious diseases, like filaria, alveolar echinococcosis, cysticercosis, and fascioliasis (1, 7).

Histology is a reliable method to confirm the diagnosis if a suitable sample of hydatid fluid/“sand” can be obtained safely by aspiration. A cyst biopsy may be more hazardous because of a higher risk of leakage, which may lead to seeding or acute anaphylaxis. In our patient, finding larvae in the hepatic cyst established the diagnosis and was important in deciding to perform the cardiac cystectomy. Although asymptomatic and with little apparent effect on cardiac function, the cyst was at high risk of rupture, given its location in the left ventricular wall. Hydatid cysts in the ventricular walls can grow toward either the epicardium or the endocardium. Subepicardial cysts grow more easily toward the pericardial cavity and can have large diameters; subendocardial cysts often have a higher potential for intracavitary growth. Surgical resection should not be delayed since medical therapy does not always result in a cure and may not prevent cyst rupture. Most cysts in the heart are resected under cardiopulmonary bypass, as was performed for our patient, to minimize the intracardiac rupture of the cyst, the hemodynamic deterioration during manipulation, or the accidental tear of the ventricular cavity (1, 7, 18, 19). Unfortunately, our patient developed mild to moderate mitral regurgitation due to prolapsed A1 and A2 segments of the anterior leaflet 12 months after surgery. It was probably related to the reduced function of the anterior papillary muscles of the left ventricle after cyst resection. This underscores the need for follow-up echocardiography to screen for valve dysfunction and residual cysts without exposure to radiation or requirement of iodine or gadolinium contrast agent.

Current guidelines for the management of cystic echinococcosis recommend that surgery be combined with adjunctive medical treatment with albendazole to sterilize the cyst and thus minimize the risk of intraoperative dissemination. Data from previous studies showed that the treatment of hydatid cysts with albendazole lowered the rate of recurrence and reduction of the size and death of the hydatid cysts (2, 20). The dose and duration of albendazole therapy vary in different reports; we treated our patient for 8 weeks with albendazole after her partial hepatic cystectomy and before her cardiac cystectomy; albendazole was continued for 2 weeks post-cardiac surgery. After 1 year, there was no evidence of relapsed hydatid disease on echocardiography and abdominal ultrasound, and the patient completely recovered.

## Conclusion

We present a rare case of *Echinococcus granulosus* from Vietnam, a very low-endemic country for hydatid disease, that involved the left ventricle and the liver. Our patient highlights the need to search for hydatid cysts outside of the liver. It also stresses the importance of 2D echocardiography and the incremental value of live/real-time 3D echocardiography over 2D imaging for the diagnosis of cardiac echinococcosis. It also confirms the roles of cardiac imaging modalities such as MDCT and MRI. Surgical excision, with adjunctive albendazole, is the best treatment for cardiac cysts to prevent catastrophic complications caused by cystic rupture, regardless of cyst dimensions. The management of hydatid disease should be based on a multidisciplinary approach involving collaboration among cardiologists, radiologists, surgeons, and infectious disease specialists.

## Data availability statement

The original contributions presented in the study are included in the article/Supplementary material, further inquiries can be directed to the corresponding author.

## Ethics statement

The studies involving human participants were reviewed and approved by Bach Mai Hospital. The patients/participants provided their written informed consent to participate in this study. Written informed consent was obtained from the individual(s) for the publication of any potentially identifiable images or data included in this article.

## Author contributions

HN, VP, and JK devised the manuscript concept. HN, VP, HP, WT, and HD belonged to the patient's management team. HN and VP performed two-dimensional/three-dimensional echocardiography. HD was the main surgeon. VP contributed to clinical, imaging, and pathological data collection. HN and VP wrote the manuscript in collaboration with JK, WT, HP, and HD. JK and WT reviewed and edited the manuscript. HN took care of revising and submitting the manuscript. All authors read and approved the final version of the manuscript.

## Supplementary material

Supplementary Video 1 3D-full volume imaging: the cyst was bulging into the left ventricle from the myocardium of the left ventricular lateral wall, moving synchronously with the cardiac cycle.

Supplementary Video 2 3D zoom imaging of the cyst from outside perspective.

Supplementary Video 3 3D zoom imaging of the cyst from inside perspective.

Supplementary Video 4 The cardiac cyst and the hepatic cyst were simultaneously depicted on MRI.
