# Supplementary material for: Concomitant intramyocardial and hepatic hydatid cysts diagnosed by multi-modality imaging: A rare case report
Source: Front Cardiovasc Med. 2022 Dec 14;9:1055000. doi: 10.3389/fcvm.2022.1055000 (PMC9795171; doi:10.3389/fcvm.2022.1055000)
Supplement: Supplementary file 5 [file Table_1.docx]

Timeline of patient clinical course

| Sep 2019 | The patient had right upper quadrant and epigastric pain. |
| --- | --- |
| 3 Nov 2019 | Hospitalization, ECG, chest X-ray, blood test (elevation of eosinophilic white blood cells), abdominal ultrasound revealed an echolucent lesion in the right lobe of the liver. |
| 5 Nov 2019 | Abdominal CT scan with contrast showed a large hepatic cyst. Brain and chest CT scans were normal. |
| 6 Nov 2019 | 2D/3D transthoracic echocardiography revealed an intramural cardiac cyst from the myocardium of the LV lateral wall. There were no mitral prolapse, no mitral annular dilation and no mitral regurgitation. |
| 7 Nov 2019 | Contrast-enhanced MDCT of the heart showed an encapsulated, rounded, non-calcified structure along the LV lateral wall. Coronary MDCT excluded coronary stenosis, coronary aneurysm. |
| 8 Nov 2019 | Cardiac MRI demonstrated an intramuscular cystic mass at the anterolateral papillary muscle attached to LV lateral wall. |
| 9 Nov 2019 | Echinococcus granulosus antibody ELISA and other parasitology tests were negative |
| 11 Nov 2019 | Surgical removal of the hepatic cyst.  Echinococcus larvae were found in the hepatic specimen.  Started the treatment with albendazole 400mg, bid, oral. |
| 7 Jan 2020 | Cardiac surgery in the cardiovascular operating room was perfomed under cardiopulmonary bypass to totally remove the cardiac cyst. Echinococcus larvae were found in the cardiac cyst fluid.  Continued the treatment with oral albendazole 200mg, bid, for two more weeks. |
| 7 Feb 2020, | One-month follow-up echocardiography and abdominal ultrasound |
| 7 July 2020 | Six-month follow-up echocardiography and abdominal ultrasound |
| 11 March 2021 | One-year follow-up echocardiography and abdominal ultrasound.  No evidence of relapsed hydatid disease on echocardiography and abdominal ultrasound.  Mild to moderate mitral regurgitation cause by anterior leaflet prolapse. |
